# Structural investigation and application of Tween 80-choline chloride self-assemblies as osmotic agent for water desalination

**DOI:** 10.1038/s41598-021-96199-6

**Published:** 2021-08-23

**Authors:** Yasamin Bide, Marzieh Arab Fashapoyeh, Soheila Shokrollahzadeh

**Affiliations:** Department of Chemical Technologies, Iranian Research Organization for Science and Technology (IROST), P.O. Box: 15815-3538, Tehran, Iran

**Keywords:** Environmental sciences, Chemistry, Engineering, Materials science

## Abstract

Forward osmosis (FO) process has been extensively considered as a potential technology that could minimize the problems of traditional water desalination processes. Finding an appropriate osmotic agent is an important concern in the FO process. For the first time, a nonionic surfactant-based draw solution was introduced using self-assemblies of Tween 80 and choline chloride. The addition of choline chloride to Tween 80 led to micelles formation with an average diameter of 11.03 nm. The ^1^H NMR spectra exhibited that all groups of Tween 80 were interacted with choline chloride by hydrogen bond and Van der Waals’ force. The influence of adding choline chloride to Tween 80 and the micellization on its osmotic activity was investigated. Despite the less activity of single components, the average water flux of 14.29 L m^‒2^ h^‒1^ was obtained using 0.15 M of Tween 80-choline chloride self-assembly as draw solution in the FO process with DI water feed solution. Moreover, various concentrations of NaCl aqueous solutions were examined as feed solution. This report proposed a possible preparation of nonionic surfactant-based draw solutions using choline chloride additive with enhanced osmotic activities that can establish an innovative field of study in water desalination by the FO process.

## Introduction

Of the most critical challenges for the growing world population are energy and water. Due to the energy need for obtaining fresh water in most cases and the water requirement for power production, the energy and water are interconnected^1^. Among the water treatment process, membrane-based techniques, and mostly reverse osmosis (RO) and forward osmosis (FO) have attracted considerable attention^2^. Unlike RO that works by external pressure, FO is an osmotically-driven membrane process with various applications like power generation, desalination, food and dairy processing, and wastewater treatment^3,4^. However, FO technology has serious problems concerning the accessibility of an efficient membrane and draw solution as the cores of the process^5^. An ideal draw solution should have the following properties: (1) low cost for producing draw solute; (2) straightforward preparation method; (3) low toxicity; (4) high osmotic pressure; (5) minimum reverse solute flux (RSF); (6) high diffusion coefficient to decrease internal concentration polarization (ICP); and (7) cost and energy-efficient draw solute recovery. However, some inconsistency may exist between these possessions^6^. For example, the small-sized draw solutes may have improved osmotic pressure and ICP, but high RSF^7^.

In this study, a mixture of a nonionic surfactant and choline chloride was employed as a draw solution. Surfactants consist of a hydrophilic and a hydrophobic group, which change the energy relationships at interfaces regarding the surface or interfacial tension^8^. Based on the charge of the groups on the hydrophilic head, surfactants are mainly categorized as anionic, cationic, nonionic, and zwitterionic^9^. The hydrophilic part can improve the interaction with an aqueous phase, while the hydrophobic fragment can interact with a relatively hydrophobic membrane to create a hydrophobic layer on the membrane surface that avoids salt transmission, and therefore decreasing reverse solute flux^10^.

At concentrations above the critical micelle concentration (CMC), the surfactant monomers aggregate to form micelles in order to minimize the free energy of the system. Micellar systems have widespread applications in food, biotechnology, chemicals, catalysis, and petroleum industries^11–13^. The interactions between micelles arising from electric double-layer repulsion can result in substantial deviation from ideal solution behavior, and consequently a remarkably elevated osmotic pressure. According to the report of Amos et al., cetylpyridinium chloride and sodium dodecyl sulfate solutions presented high osmotic pressure confirming the non-ideal intermicellar interactions^14,15^.

Various micellar draw solutions have been recently developed due to their unique features which affect the FO performance^10,15,16^. Among the different surfactants used as draw solutions, nonionic surfactants have not been investigated probably due to their low osmotic pressure and consequently low water flux. The additives can change the phase and micellar behavior of nonionic surfactants based on the solubilization places in the micelle. In this work, Tween 80 as a nonionic surfactant with low cost and low toxicity was chosen to study the nonionic surfactant-based draw solution. The influence of additives to surfactants for various applications has been reported in the literature. For example, Łuczak et al. studied the micelle formation of Tween 20 in imidazolium ionic liquids for different potential applications^17^. Deng et al. investigated the interactions in the solubilized thymol in Tween 80 micelle. They suggested the solubilization mechanism affected the antioxidant activity^18^. In this study, the interaction of Tween 80 with choline chloride as an inexpensive, largely available, and nontoxic hydrogen bond receptor is examined and their application as the osmotic agent in the FO process was explored.

## Experimental

### Materials

Tween 80 (Polysorbate) (critical micelle concentration of 0.014 g L^‒1^) and choline chloride (CC) were obtained from Merck Co. Deionized water (DI) was used in the FO tests.

### Instruments and characterization

Gel permeation chromatography (GPC) analysis was performed using a Shimadzu LC-20A instrument with an Ultrahydrogel 250 column and refractive index detector. The polyethylene glycol was employed as standard. The mobile phase was 0.1 M NaNO_3_ in water at a flow rate of 1 mL min^‒1^. To determine the functional groups of as-prepared self-assembly material, FTIR analysis was performed using a Thermo-Nicolet AVATAR 350 spectrometer. An STA 1500 instrument in an N_2_ atmosphere with a heating rate of 10 °C min^–1^ was employed to obtain a thermogravimetric analysis (TGA) curve. DLS analysis was performed with a Malvern Nano ZS (red badge) ZEN 3600 to identify the size distribution of Tween 80-CC self-assembly. The ^1^H NMR spectra were obtained by a BRUKER DRX-300 AVANCE spectrometer, using CDCl_3_ as the solvent. The surface tension of the samples was measured using a Jikan CAG-20 device at 23 °C. The conductivity meter (Hach HQ14d) was utilized to measure the conductivity of solutions at 25 °C. The osmotic pressure was determined according to the relative air humidity measured by a sensor (SHT10, Sensirion) connected to a double-layered jacket glass vessel^19,20^. The temperature control was accomplished through a heat transfer solution circulated in the outer layer.

### Synthesis of Tween 80-CC self-assembly

**Scheme 1:**
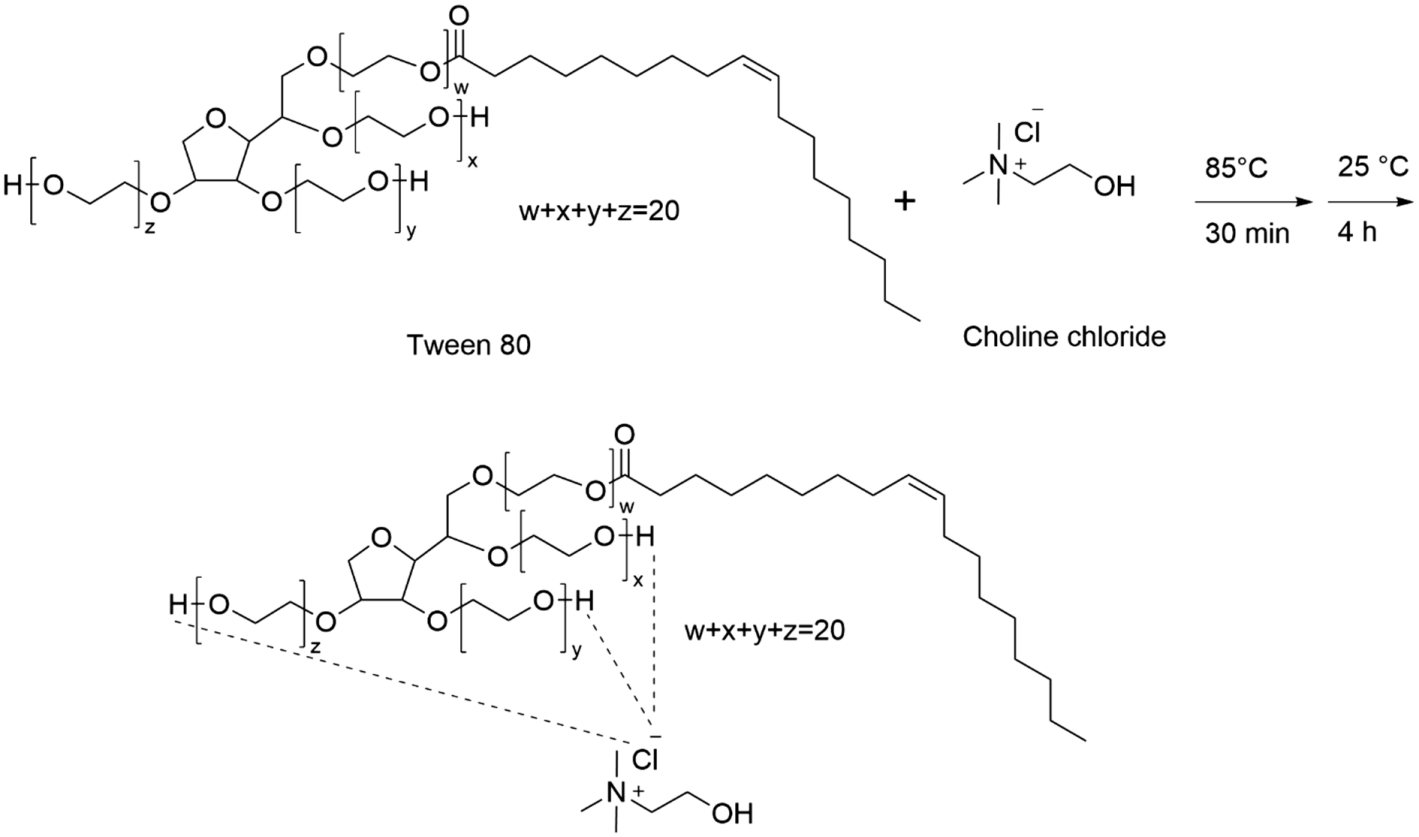
Synthesis of Tween 80-CC self-assembly.

The Tween 80-CC self-assembly was simply prepared by mixing Tween 80 and CC with a determined molar ratio (Scheme 1). The mixture was heated to 85 °C for 30 min and continuously stirred for 4 h until a homogeneous liquid was gained at room temperature. Various molar ratios of Tween 80:CC including 1:1, 1:2, and 2:1 (namely Tween 80_1_-CC_1_, Tween 80_1_-CC_2_, and Tween 80_2_-CC_1_) were synthesized to examine the influence of the composition on the osmotic pressure of the solutions.

### FO experiments

To evaluate the efficiency of as-prepared Tween 80-CC self-assemblies as an osmotic agent, a lab-scale FO setup consisting of a cross-flow cell, pumps, pressure gauges, and flow meters were employed. A cellulose triacetate (CTA) flat sheet membrane was employed and wetted in DI water before use. In the FO tests, the water flux was assessed using DI or saline water as feed and Tween 80-CC self-assemblies as a draw material in FO or PRO mode with a membrane active layer facing the feed solution or active layer facing the draw solution, respectively. The experiments were accomplished at a flow rate of 1.2 L min^−1^ for both feed and draw sides to remove the impact of external concentration polarization. The pressure inside the draw side was atmospheric pressure. The temperature was set at 25 ± 1 °C throughout the tests. After each FO test, the system was washed with DI water. The water flux (L m^−2^ h^−1^, LMH) from the feed to the draw solution through the membrane was determined by measuring the increase of volume of the draw solution during the operation test according to Eq. S1. The RSF (g m^−2^ h^−1^, gMH) was calculated according to Eq. S2 by measuring the feed solution conductivity. The concentration of feed solution at a determined time was obtained according to the concentration–conductivity calibration curve. The water permeance was calculated based on the normalized water flux by osmotic pressure (LMH bar^‒1^).

### Regeneration of draw solution

A low energy method was examined for the regeneration of micelles from the diluted draw solution. This technique includes microfiltration (MF) using a polyethersulfone membrane of 150 kDa molecular weight cut off (MWCO) prepared from 3 M company, Germany.

## Results and discussion

### Osmotic pressure

For the sustainable FO process, the draw solution should give significant osmotic pressure across the membrane even after dilution. To understand the colligative properties of Tween 80-CC self-assemblies, the osmotic pressure as the vital contributing parameter was measured at different compositions and concentrations. Due to the very low osmotic pressure of nonionic surfactants, their use as an osmotic agent was restricted^21^. As expected, Tween 80 represented a very low osmotic pressure (Table 1). The effect of choline chloride addition to the Tween 80 with various ratios on the osmotic pressure of as-prepared self-assemblies was summarized in Table 1. At the same mass concentration of 247 g L^−1^, the Tween 80_1_-CC_2_ exhibited the highest osmotic pressure (i.e., 155.7 atm). Therefore, the as-prepared Tween 80_1_-CC_2_ was characterized by various analyses.

**Table 1:** The osmotic pressure calculated from air humidity measurements as a function of concentration and compositions of Tween 80-CC self-assemblies at 25 °C.

Entry	Concentration (g L^−1^)	Osmotic pressure (atm.)
Tween 80	166	2.7
CC	82	19.1
Tween 80_1_-CC_2_	123	38.3
	182	78.2
	247	155.7
	309	176.9
Tween 80_1_-CC_1_	247	94.2
Tween 80_2_-CC_1_	247	45.7
NaCl	60	49.6

### Characterization of Micelles

#### FTIR analysis

**Figure 1:**
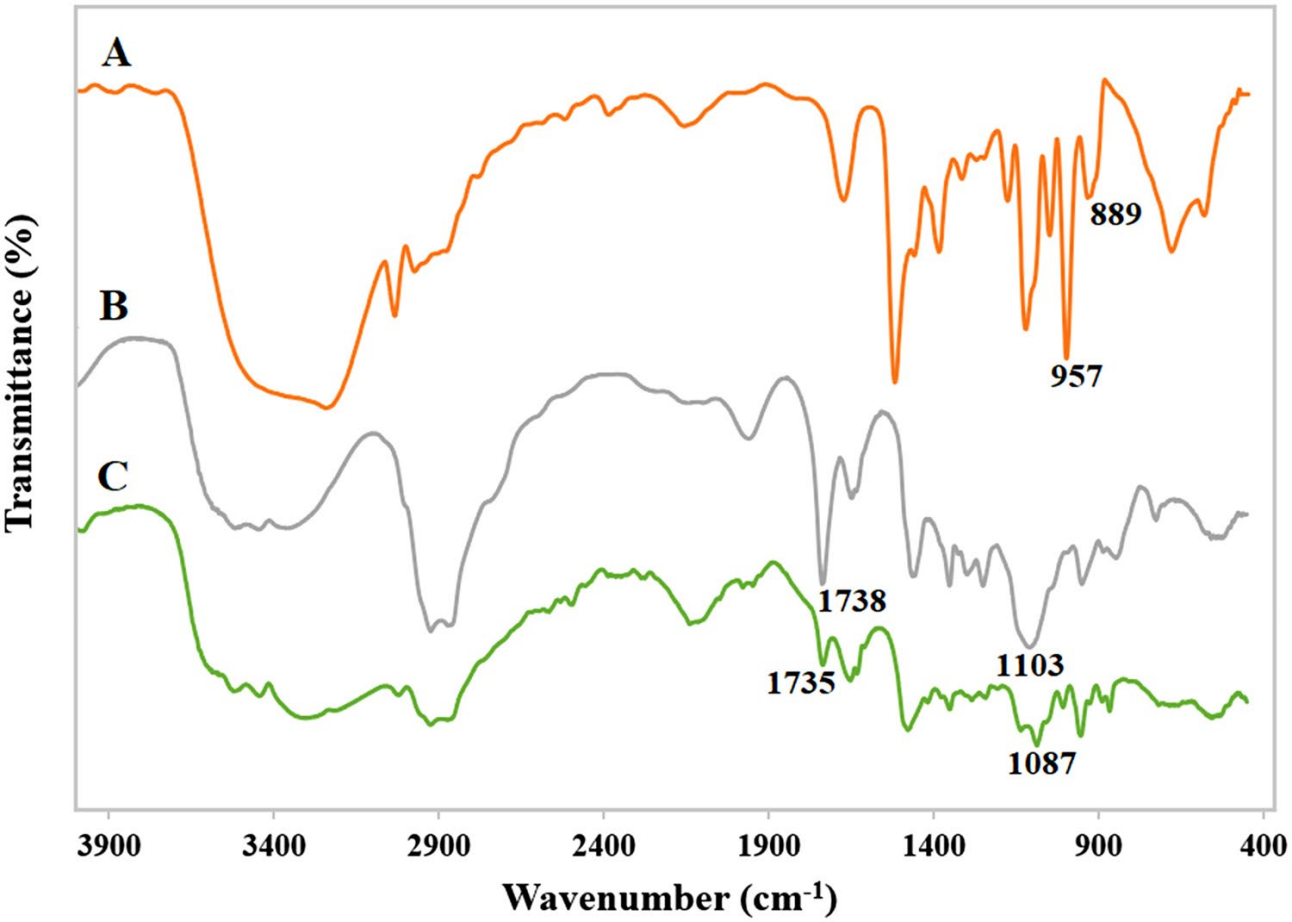
FTIR spectra of CC (**A**), Tween 80 (**B**), and Tween 80_1_-CC_2_ self-assemblies (**C**).

FTIR analysis of Tween 80_1_-CC_2_ and the components, CC and Tween 80, was performed to evaluate the interactions between functional groups of CC and Tween 80 (Fig. 1A–C). The FTIR spectrum of CC displays the characteristic bands at 889, 957, and 1021 cm^‒1^ allocated to the C–N^+^ stretching, and C–C–O symmetric and C–C–O asymmetric vibrations of choline chloride, respectively^22^. A broad hydroxyl peak in the 3100–3700 cm^‒1^ range was also observed (Fig. 1A). In the FTIR spectrum of Tween 80, a broad and strong band was observed in the range of 3100–3700 cm^‒1^ due to the stretching vibrations of O–H. The bands at 1103 and 1738 cm^‒1^ are respectively ascribed to the asymmetric C–O and C=O stretching vibrations (Fig. 1B). The spectrum of the Tween 80_1_-CC_2_ is roughly an overlap of Tween 80 and CC peaks. Compared to Tween 80, the band at 3100–3700 cm^‒1^ was shifted towards the lower wavenumber and changed to the broader band in the Tween 80_1_-CC_2_ spectrum. This alteration suggests the formation of hydrogen bonds between Tween 80 and CC^23^. Moreover, after the addition of CC, the absorption bands corresponded to the C–O and C=O stretching vibrations of Tween 80 red-shifted to 1087 and 1735 cm^‒1^, respectively, which further confirms the hydrogen bond interaction between Tween 80 and CC (Fig. 1C)^24,25^.

#### ^1^H NMR analysis

**Figure 2:**
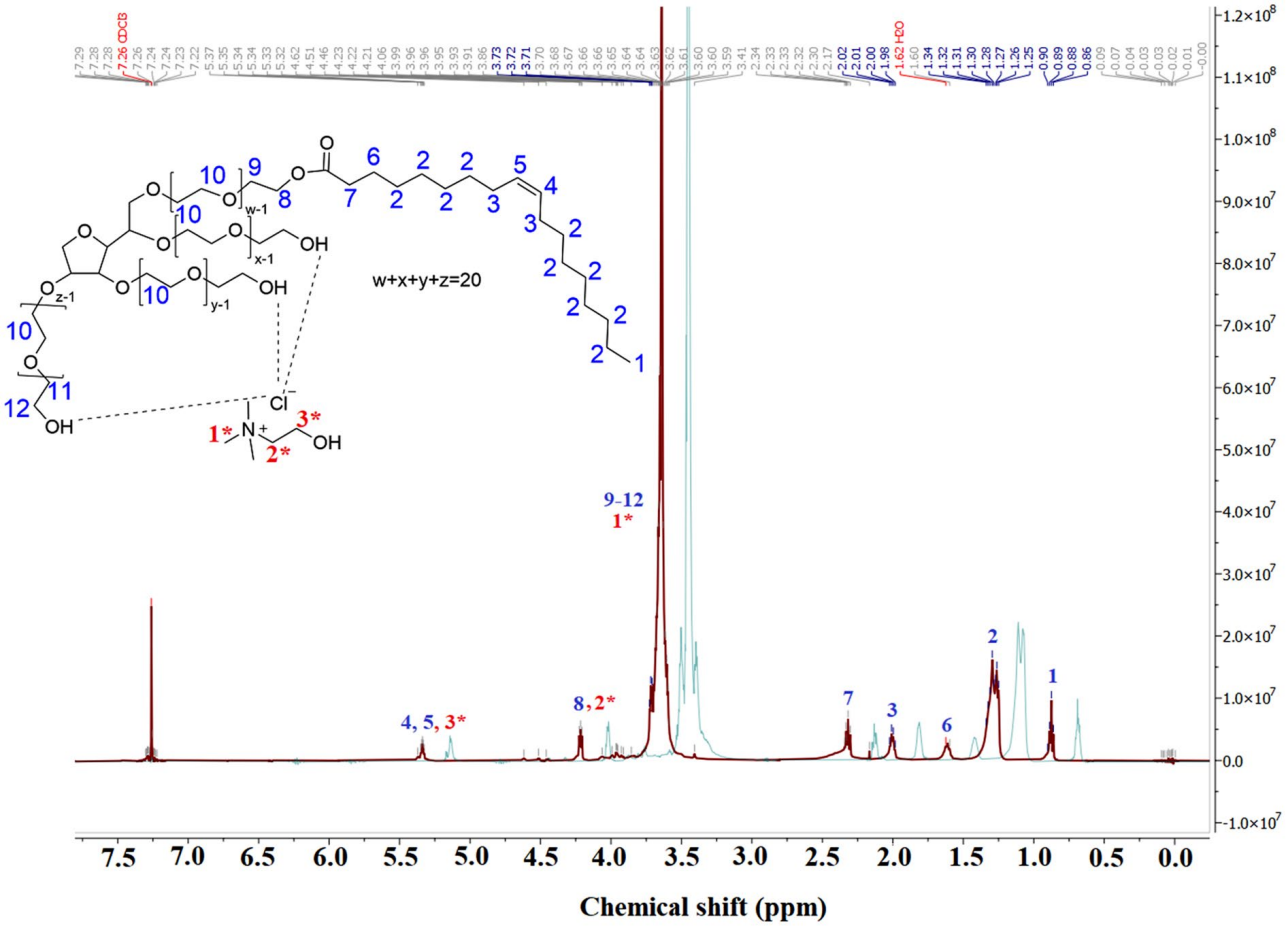
^1^H NMR spectra of Tween 80_1_-CC_2_ self-assembly, and Tween 80 (shadow) in CDCl_3_.

The ^1^H NMR analysis is a powerful technique to identify the localization of molecules in the surfactant micelles^26^. The ^1^H NMR spectrum of Tween 80_1_-CC_2_ self-assemblies was presented in Fig. 2. The shadow of Fig. 2 is the ^1^H NMR spectrum of Tween 80. The CC showed three ^1^H NMR peaks in 3.29, 3.56, and 4.02 ppm attributed to C**H**_3_, C**H**_2_-N^+^, and C**H**_2_-O groups, respectively. Due to the red shift of peaks, the CC peaks were probably overlapped with the peak of 3.7, and 4.23 peaks of Tween 80_1_-CC_2_ self-assemblies. As observed, eight proton peaks of the Tween 80 also exist in the spectrum of Tween 80-CC and all of them considerably shifted down-field after the addition of CC. The shifted protons indicated that all of the Tween 80 protons were affected by the interactions between Tween 80 and CC including hydrogen bond and Van der Waals' force^18^. Therefore, we propose that the CC molecules interact with Tween 80 micelles through different functional groups.

#### TG analysis

**Figure 3:**
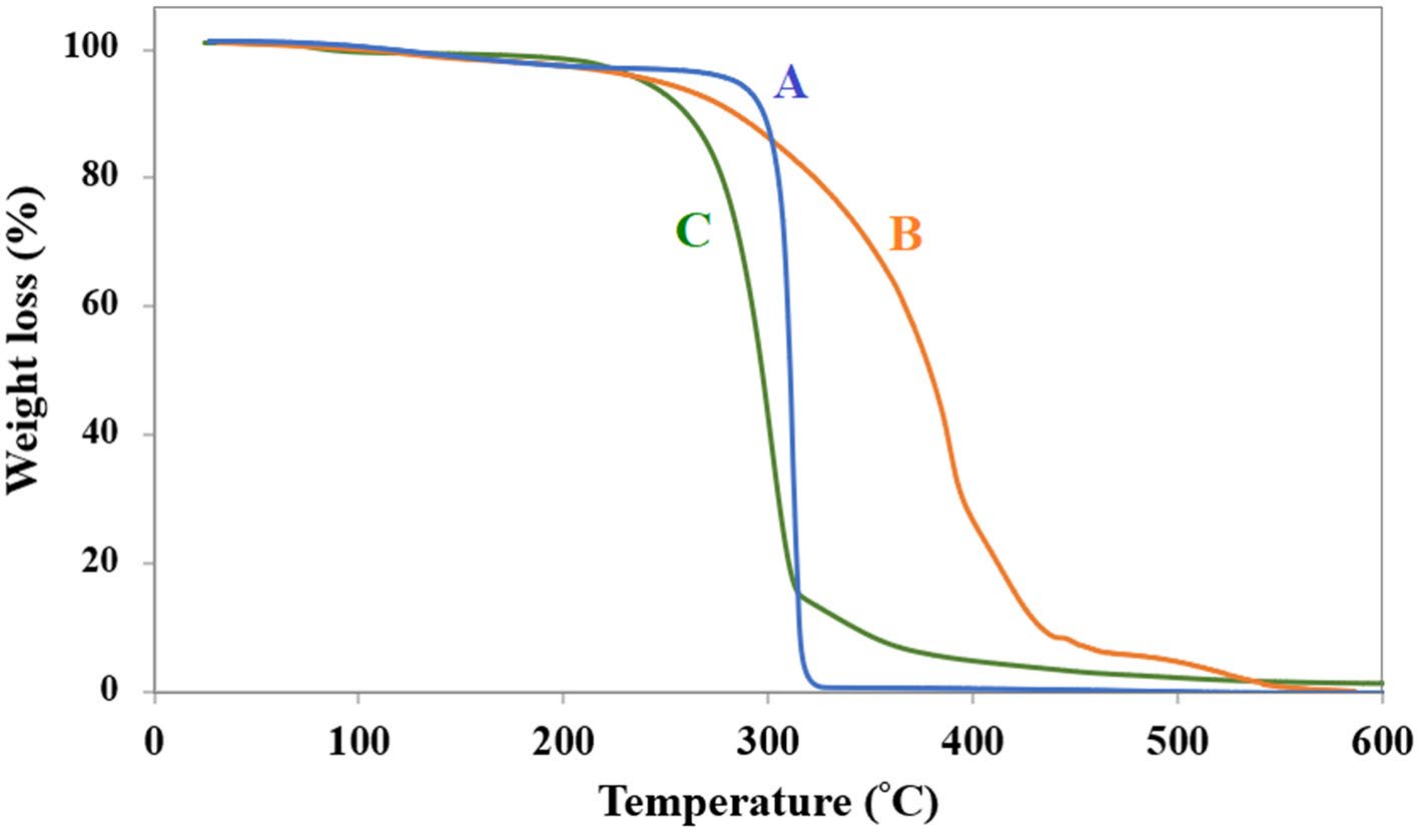
TGA curve of CC (**A**), Tween 80 (**B**), and Tween 80_1_-CC_2_ self-assembly (**C**).

The thermal decomposition temperatures of the Tween 80_1_-CC_2_ mixture and its components were measured by TGA analysis as shown in Fig. 3. The maximum temperature at which the mixture can preserve its liquid state without decomposition is a vital feature. The as-prepared mixture showed an onset decomposition temperature at around 230 °C (Fig. 3C), which is between those of their pure constituents (Fig. 3A,B). The choline chloride and Tween 80 begin to decompose at around 270 and 200 °C, respectively^27–29^.

#### GPC analysis

GPC analysis was utilized to determine the molecular weight of the Tween 80_1_-CC_2_ self-assembly. The weight average molecular weight (M_w_) and number average molecular weight (M_n_) were 1595 and 1566 g mol^‒1^, respectively (Figure S1). According to the GPC results, the as-prepared self-assemblies included 1:2 molar ratios of Tween 80:CC that is in good agreement with the experimental procedure using the 1:2 molar ratios. This confirms the complete interaction of Tween 80 with CC without any unreacted and free molecules which may cause reverse micelle flux through the FO membrane. Moreover, the polydispersity index (PDI) was 1.02 which exhibits that the molecular weight distribution of the Tween 80_1_-CC_2_ self-assemblies was completely homogeneous.

### Surface tension measurements

**Figure 4:**
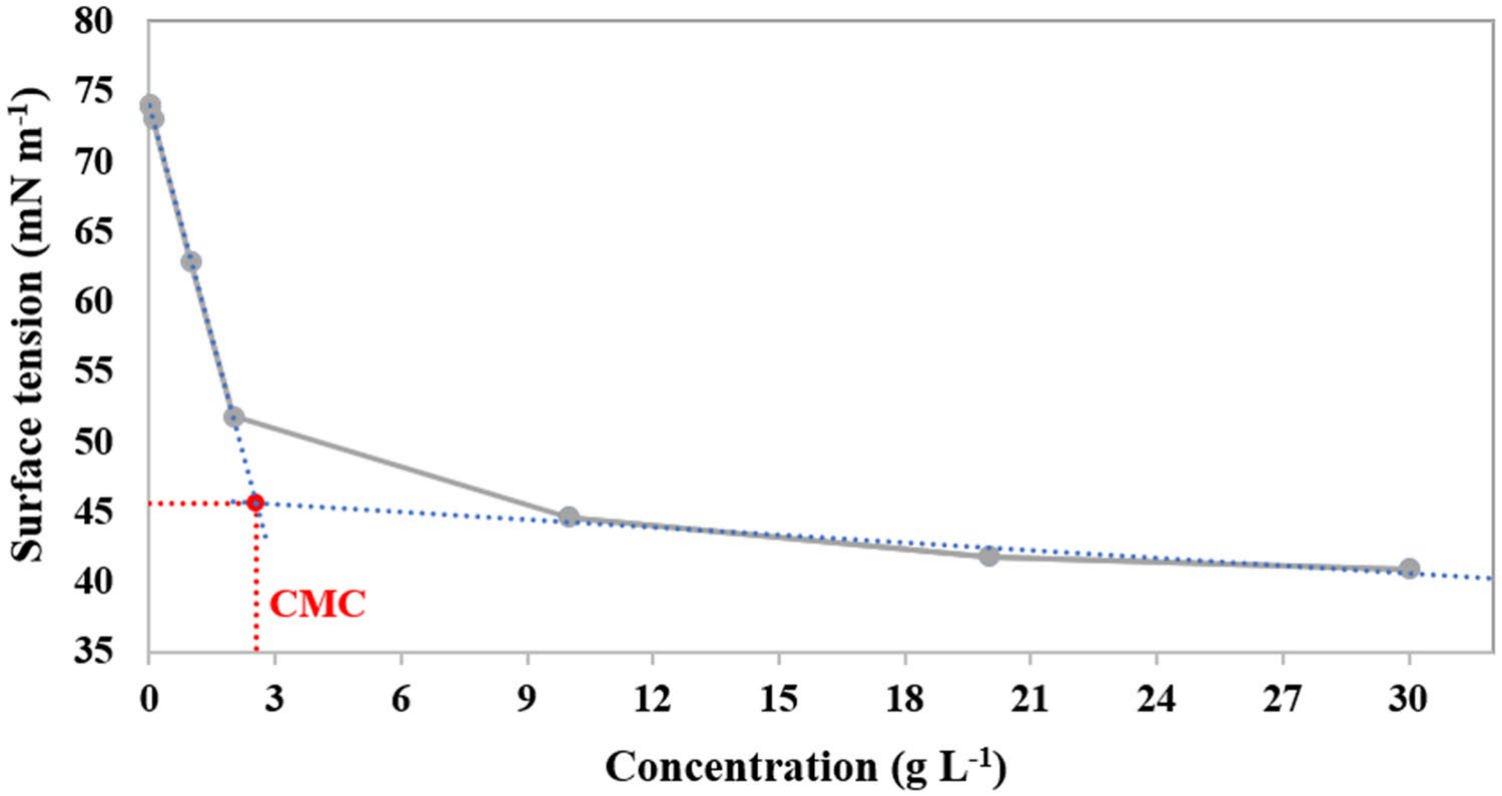
Surface tension isotherms (air–water interface) of Tween 80_1_-CC_2_.

To evaluate the surface activity of Tween 80-CC self-assemblies, the surface tension isotherms were provided (Fig. 4). The adsorption at air–water interface and micellization of Tween 80-CC self-assemblies were designated by the surface tension assessment versus Tween 80_1_-CC_2_ concentrations. Based on the results, Tween 80 in the CC solutions remains surface-active due to the variation of surface tension with concentration. As the concentration of Tween 80-CC aqueous solution increased, the surface tension was gradually decreased, showing adsorption of the surfactant molecules at the air–water interface. At the CMC, a discontinuity was existed in the *γ*-log *c* curve, indicating the self-assembling of Tween 80_1_-CC_2_ to form micelles. Therefore, the CMC was obtained as 2.5 g L^‒1^. Compared to the CMC value of Tween 80 aqueous solution (0.014 g L^‒1^), the CMC was increased considerably representing salting in of the surfactants^17^. The hydrophilic part of Tween 80 is a kind of polyether that can act as polydentate ligands for CC through hydrogen bonds. Since the binding of CC weakens the hydrophobic interaction of Tween 80 with water, increases its solubility in CC solutions above that in water, and consequently the CMC increases^30^. As the hydrophilicity of the as-prepared material is increased, it is expected that its osmotic activity also enhances in the FO process.

### DLS analysis

**Figure 5:**
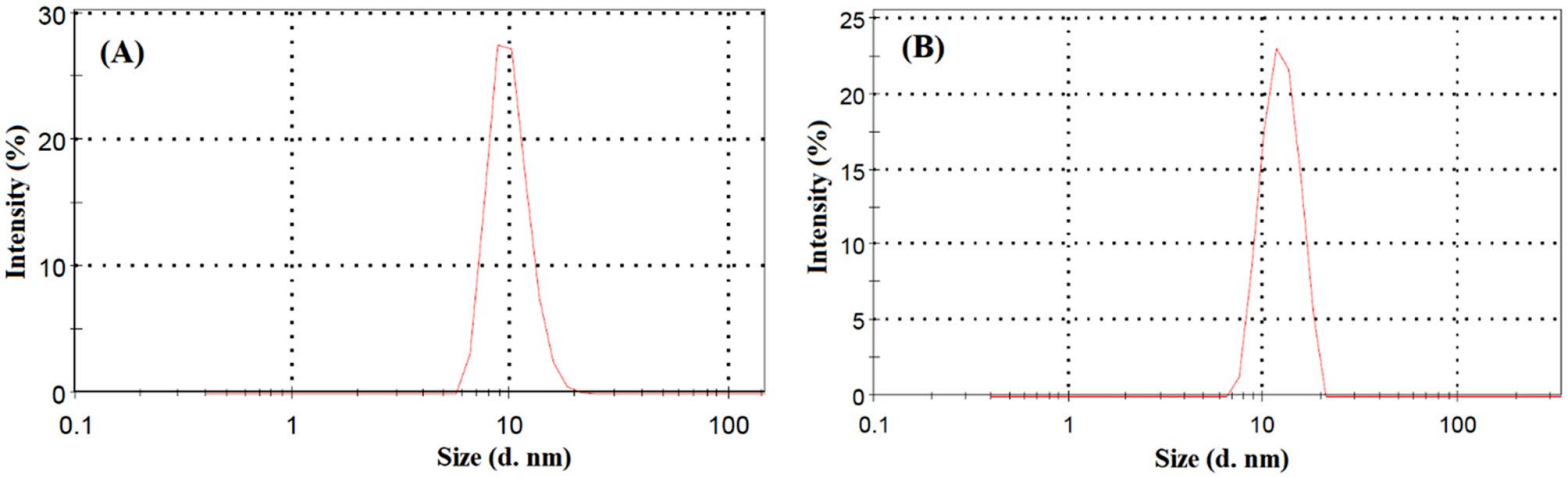
Histograms representing micellar size distribution of Tween 80 (**A**) and Tween 80_1_-CC_2_ self-assemblies (**B**) with 2.5 g L^‒1^ concentration at 25 °C.

DLS analysis is a valuable procedure for investigating hydrodynamic radii of nano-scale particles. DLS generally determines the time-dependent variations in the intensity of scattered light from a colloidal solution. We employed DLS analysis to evaluate the effect of CC addition on the micelle size (hydrodynamic diameter) of Tween 80 micelles. The average hydrodynamic diameter of aqueous solution of Tween 80 micelle at CMC was obtained to be 9.95 nm (Fig. 5A), which is in good agreement with literature^31^. Histograms representing micellar size distribution of Tween 80_1_-CC_2_ mixtures have been shown in Fig. 5B. According to the results, the hydrodynamic diameter of Tween 80_1_-CC_2_ mixtures was found to be 11.08 nm. The high-intensity signals designate the monodisperse nature of micelles in an aqueous solution. Compared to single Tween 80 micelles, the mixture of CC and Tween 80 represented a relatively higher average diameter. The diameter increasing after the addition of molecules to the micelles has also been reported in literature^32,33^. The results exhibited that CC has been successfully incorporated in Tween 80 micelles and consequently more stretched structure.

### Optimization of composition

**Figure 6:**
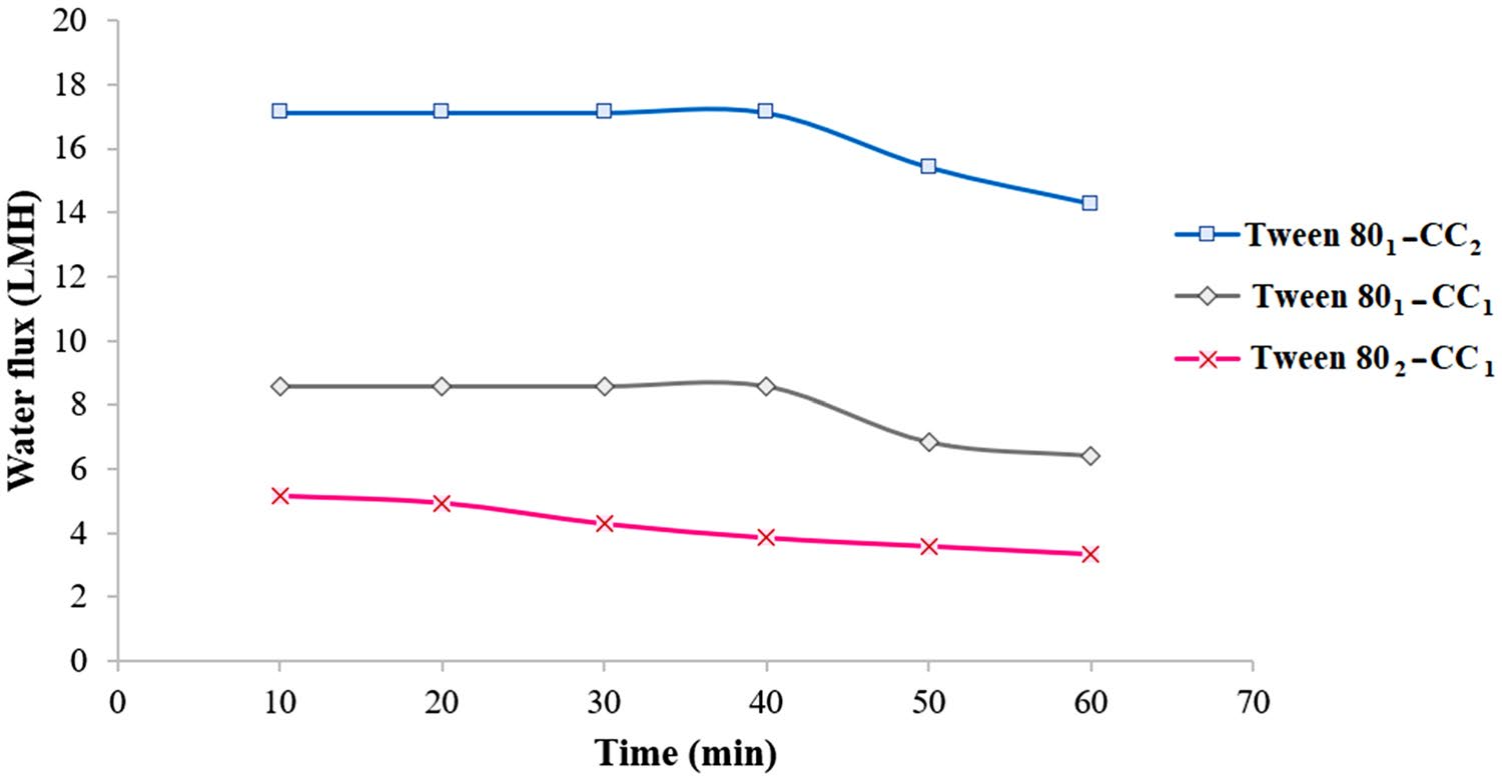
Average water flux using 247 g L^‒1^ (0.15 M) Tween 80_1_-CC_2_, Tween 80_1_-CC_1_, and Tween 80_2_-CC_1_ self-assemblies as draw solution and DI water feed solution under the PRO mode at 25 °C.

The impact of Tween 80 to CC molar ratios of the as-prepared mixture on the PRO performance was explored using 1:2, 1:1, and 2:1 molar ratios of Tween 80:CC. The solubilization of CC in the Tween 80 was easily carried out at 80 °C to form a transparent liquid. To have a correct comparison, different molar ratios were employed as draw solution under the PRO mode with similar concentrations. The water flux of different compositions as a function of time with DI water as feed solution was plotted in Fig. 6. The Tween 80_1_-CC_2_ showed the best PRO performance with 14.29 LMH average water flux which is due to the enhanced hydrophilicity of self-assembly structure containing two CC molecules binding to Tween 80.

To better understanding the effect of composition, the normalized water flux by osmotic pressure (i.e., water permeance, LMH bar^‒1^) of three samples were calculated. The Tween 80_1_-CC_2_, Tween 80_1_-CC_1_, and Tween 80_2_-CC_1_ self-assemblies showed the water permeance of 0.1, 0.08, and 0.09 LMH bar^‒1^, respectively. So, the Tween 80_1_-CC_2_ was chosen as the optimum structure for further assessments.

### Water flux performance using Tween 80_1_-CC_2_ self-assemblies draw solute

The experiments with DI water feed established the advantages of the Tween 80-CC self-assemblies compared to the single Tween 80 and CC draw solutions. While the process using the single components gave no water flux, the tests using the Tween 80-CC self-assemblies attained high water flux. For example, at the draw solution concentration of 247 g L^‒1^ (0.15 M), the PRO experiment with the Tween 80_1_-CC_2_ self-assemblies draw solution achieved a water flux of 14.29 LMH.

**Figure 7:**
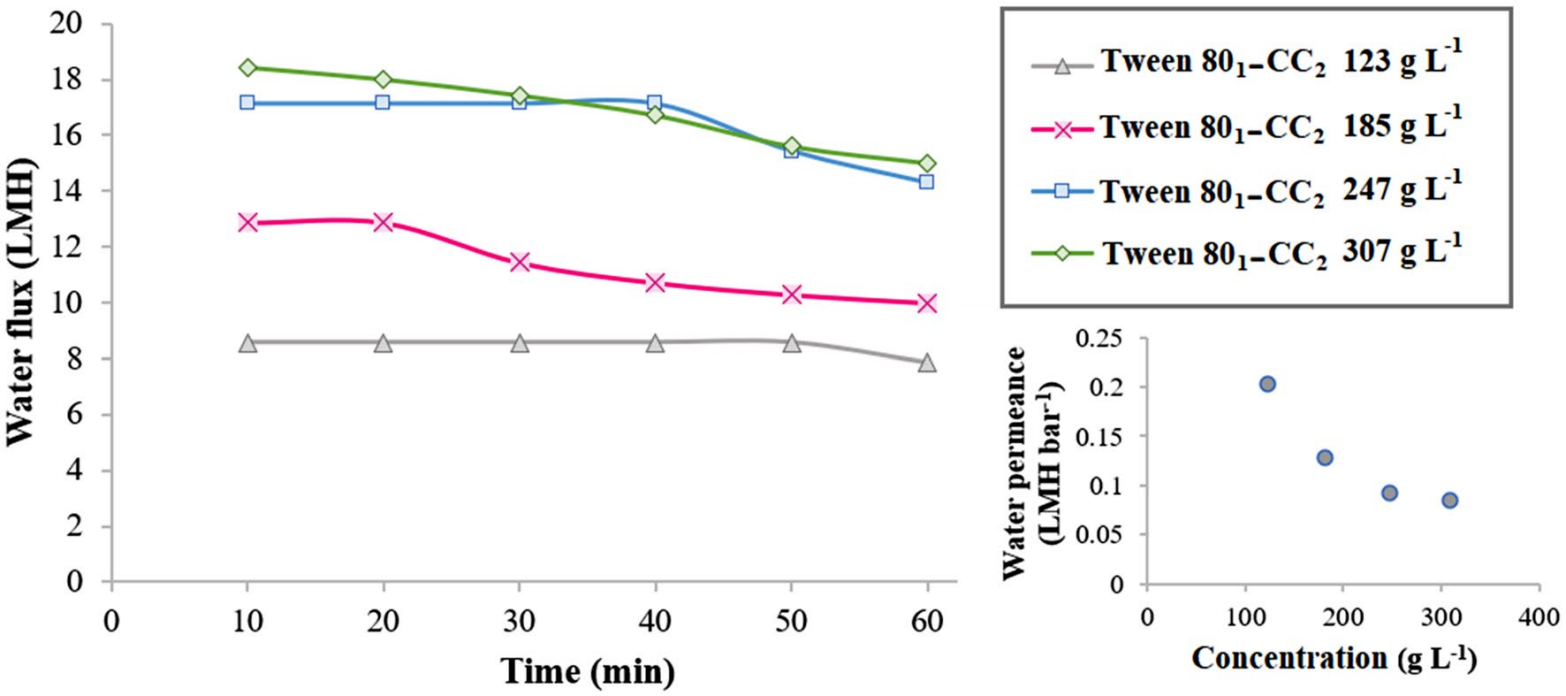
Average water flux versus time using 123, 185, 247, and 307 g L^‒1^ Tween 80_1_-CC_2_ self-assemblies as draw solution and DI water feed solution under the PRO mode at 25 °C, inset: water permeance *vs.* concentration of draw solution.

Figure 7 exhibited the time-dependent water flux using different concentrations of Tween 80_1_-CC_2_ self-assemblies (185–307 g L^‒1^) as draw solution and DI water as feed solution in the PRO mode. According to the classical solution-diffusion model, the higher water flux was obtained using more draw solution concentration because of the elevated driving force (osmotic pressure difference in this case) through the membrane^34–36^. The influence of the Tween 80-CC micelles concentration in the draw solution on the water permeance was also investigated (Fig. 7, inset). As the Tween 80-CC micelles concentration increased, the average water flux across the membrane increased due to the increase in the driving force, which lead to increase in concentration polarization and a decrease in water permeance^37,38^.

### Desalination process

**Figure 8:**
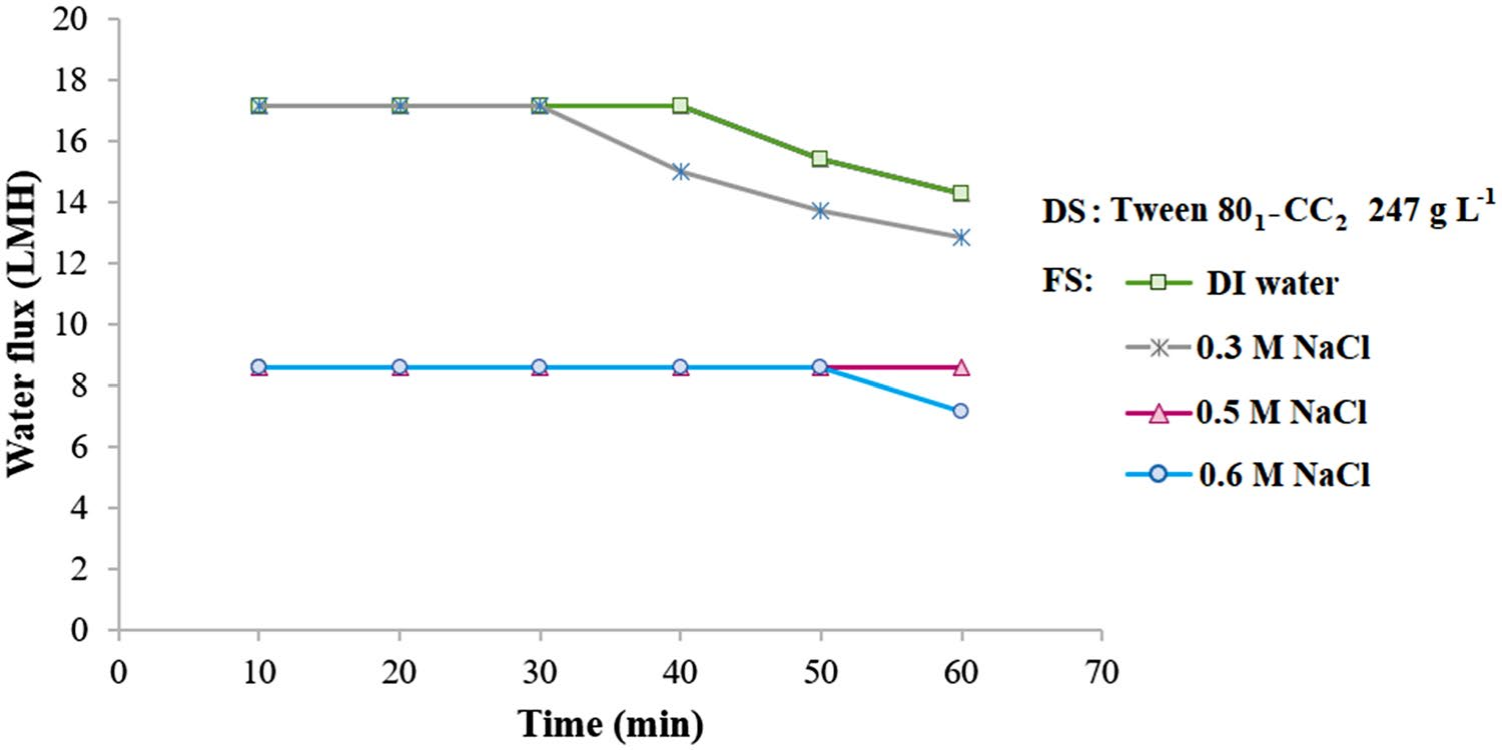
Average water flux using 247 g L^‒1^ Tween 80_1_-CC_2_ self-assemblies as draw solution and DI water, 0.3, 0.5, and 0.6 M NaCl feed solutions under the PRO mode at 25 °C.

The osmotic ability of the Tween 80_1_-CC_2_ self-assembly solution was explored using different concentrations of NaCl solutions (0–0.6 M) as the feed under the PRO mode (Fig. 8). The Tween 80_1_-CC_2_ self-assemblies not only show high water flux for DI water but also for saline water up to 0.6 M. With increasing the concentration of feed solution from 0 to 0.3 M, the initial water flux was surprisingly unchanged with a relatively small flux decline during the process which seems unusual based on the osmolality difference between the feed and draw solutions. Experiments were repeated three times to verify the results. The increase in the feed solution concentration from 0.3 to 0.5 M caused a sharp decrease in the water flux, but additional increasing the feed concentration led to more slowly the reduced water flux.

**Figure 9:**
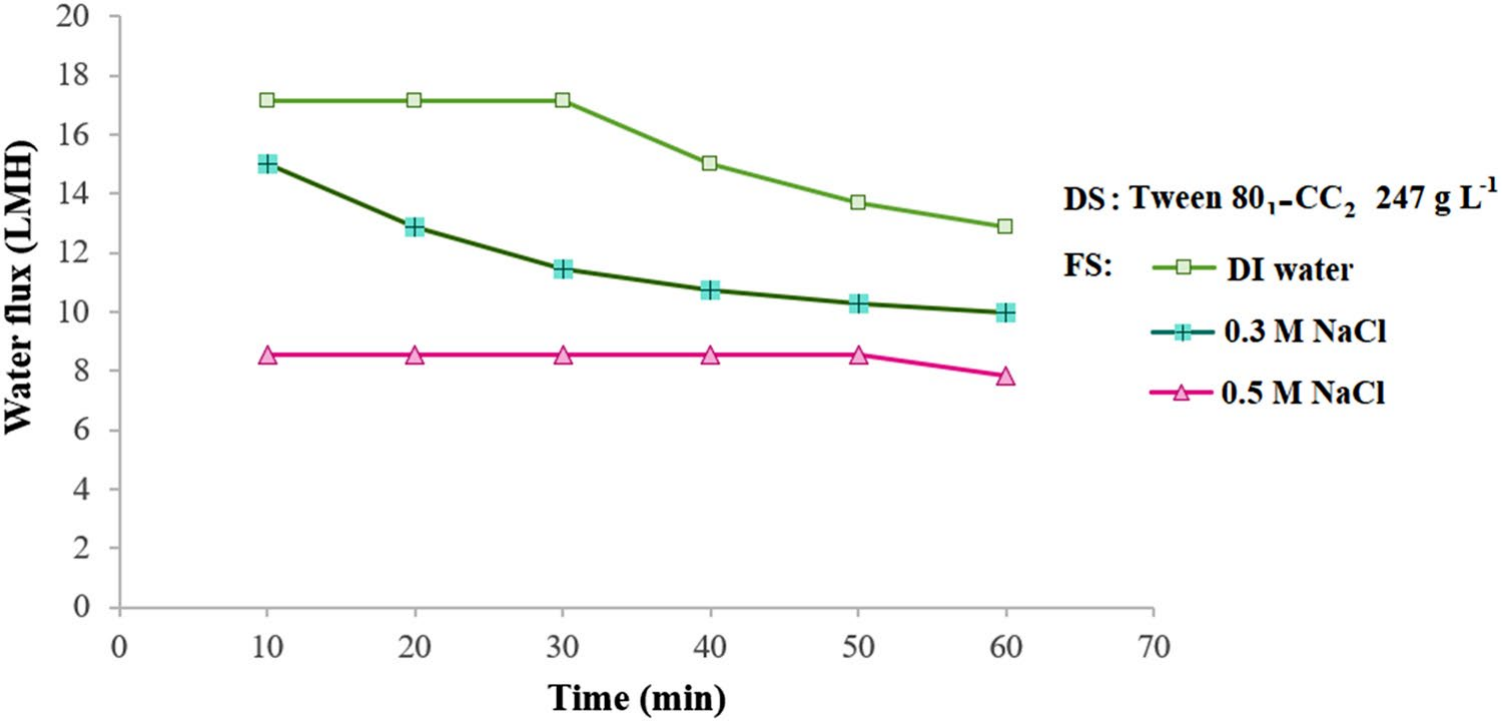
Average water flux using 247 g L^‒1^ Tween 80_1_-CC_2_ self-assemblies as draw solution and DI water, 0.3, and 0.5 M NaCl feed solutions under the FO mode at 25 °C.

One of the most significant aspects affecting the FO process is membrane orientation^39^. The influence of membrane orientation on the water flux was investigated using Tween 80_1_-CC_2_ self-assemblies draw solute (Figs. 8, 9). The FO mode results in the same water flux but more flux decline because of the decrease in osmotic potential, while in the PRO mode which the active layer facing the draw solution shows milder flux decline. In the desalination process, 0.3 and 0.5 M NaCl respectively exhibited the average water flux of 10.0 and 7.8 LMH, under the FO mode and 12.8 and 8.4 LMH under the PRO mode.

The desalination experiments using Tween 80_1_-CC_2_ self-assemblies in the FO process under both FO and PRO modes were repeated three times to ensure the results.

### Reverse Micelle flux

**Figure 10:**
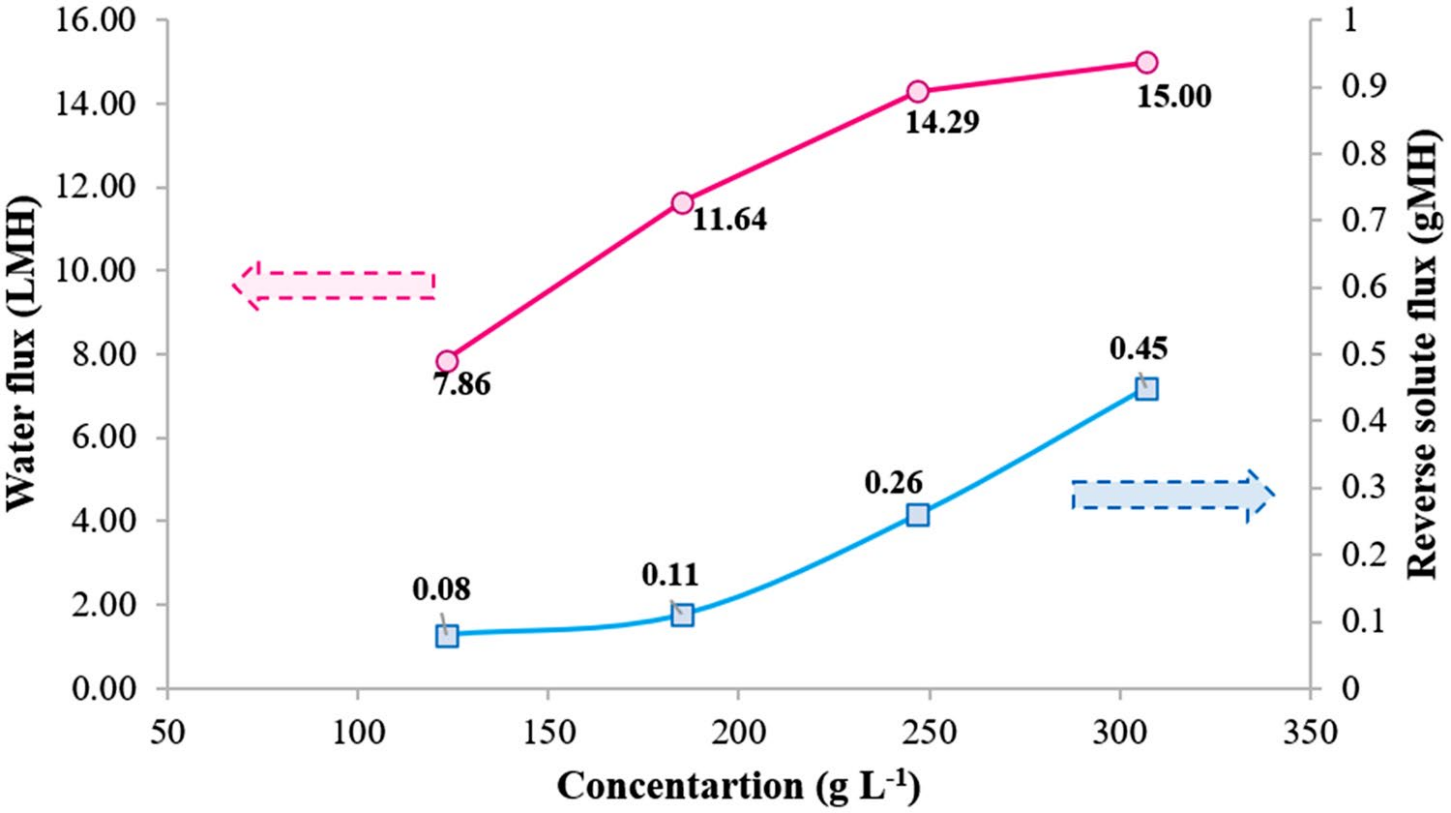
Average water flux and reverse solute flux using 123, 185, 247, and 307 g L^‒1^ Tween 80_1_-CC_2_ self-assemblies as draw solutions and DI water feed solution under the PRO mode at 25 °C.

The quality of feed water greatly depends on the reverse solute flux which rises the cost of the FO process^40^. The reverse leakage of Tween 80_1_-CC_2_ self-assembly was studied regarding its flux through the membrane, J_S_ with unit gMH. According to the results, RSF caused by 247 g L^‒1^ micellar draw solution was only 0.26 gMH (Fig. 10). The Tween 80 and CC combination can produce large-sized particles that can be simply reserved in the draw side of the FO setup as well as the great features of the micelles. Besides, the hydrophobic interactions between tail groups of Tween 80 with membrane probably make an additional layer on the membrane surface which inhibits ions from escaping through the membrane and consequently reduces RSF.

Several conventional draw solutes had been compared with this work in terms of draw solution concentration, reverse solute flux (J_s_), and specific reverse salt diffusion (J_s_/J_w_) (Table 2). The comparison showed better performance of Tween 80_1_-CC_2_ self-assemblies in terms of J_w_, J_s_ and J_s_/J_w_.

**Table 2:** FO performance of Tween 80_1_-CC_2_ self-assemblies in comparison with several conventional draw solutes using DI water as feed solution and CTA membrane.

Entry	Draw solute	Draw solution concentration (g L^−1)^	J_w_ (LMH)	J_s_ (gMH)	J_s_/J_w_ (g L^−1^)
1	NaCl	50.8	12.13	9.10	0.750
2	NH_4_HCO_3_	83.4	10.25	20.60	2.010
3	CaCl_2_	62.3	11.59	9.50	0.820
4	Na_2_SO_4_	127.3	9.39	3.10	0.330
5	MgSO_4_	141.3	5.71	1.20	0.210
6	This study	247	14.24	0.26	0.018

Experimental data for the five first rows were taken from^41^.

### Recycling draw solute

To recycle the diluted draw solution for the next run and decrease the operation cost of the FO process, the Tween 80-CC self-assemblies were regenerated by microfiltration (MF), which is a highly efficient low-pressure-driven process. After a PRO process using 247 g L^−1^ Tween 80_1_-CC_2_ self-assemblies as draw solutions and DI water feed solution at 25 °C, diluted draw solution was treated by MF process using a polyethersulfone membrane of 150 kDa MWCO. According to the results, the draw solution after the FO test was recovered by three runs of MF receiving much lower conductivity (1.1 mS cm^-1^) than that of the diluted draw solution (25.7 mS cm^-1^) with high water flux of 107.1 LMH. The conductivity of concentrate after three runs of MF were measured to be 31.7 mS cm^-1^, which is comparable to the initial draw solution (33.5 mS cm^-1^). Therefore, using the MF process, more than 96% rejection efficiency was attained.

## Conclusion

A micelle comprising Tween 80 nonionic surfactant and CC suggests the advantages of CC near to the micelles as well as ionic nature for osmotic activity. The optimum molar ratio of Tween 80 to choline chloride was obtained as 1:2. Various analyses including FTIR, ^1^H NMR, TGA, and GPC were provided to carefully characterize the Tween 80_1_-CC_2_ mixture. Besides, the surface tension and DLS analysis were carried out to further inquiry of the micellar structure and precise description of this interesting structure. The micelle size increased with the addition of CC due to the introduction of polar groups. The results were supported by the CMC value and explained based on hydrophilic interaction. The osmotic activity of as-prepared Tween 80-CC self-assemblies with several concentrations was examined as a draw solution for the FO process. The high water flux for desalination of saline water was obtained both in the FO and PRO modes. According to the experiments, very low RSF was attained using Tween 80_1_-CC_2_ self-assemblies as draw solution probably due to the large size as well as hydrophobic interaction of tail groups of Tween 80 with the membrane. Moreover, the draw solute could be easily recovered by three runs of MF with the solute rejection of 96%.

## Supplementary Information

Supplementary Information 1.
