# Supplementary material for: Structural investigation and application of Tween 80-choline chloride self-assemblies as osmotic agent for water desalination
Source: Sci Rep. 2021 Aug 23;11:17068. doi: 10.1038/s41598-021-96199-6 (PMC8382744; doi:10.1038/s41598-021-96199-6)
Supplement: Supplementary file 1 — Supplementary Information 1. [file 41598_2021_96199_MOESM1_ESM.docx]

**Supporting Information**

**Structural Investigation and Application of Tween 80-Choline Chloride Self-Assemblies as Osmoting Agent for Water Desalination**

Yasamin Bide, Marzieh Arab Fashapoyeh, Soheila Shokrollahzadeh*

Department of Chemical Technologies, Iranian Research Organization for Science and Technology (IROST), P.O. Box: 15815-3538, Tehran, Iran.

- **Water flux**

$$J_{v}= \frac{V_{tn}-V_{t0}}{A(t_{n}-t_{0})} (S1)$$

Where J_v_ is volumetric water flux, V_tn_ and V_t0_ (L) are the volume levels of draw solution at the time $t_{n}$ and $t_{0}$ (h), respectively, during the FO test and A (m^2^) is the effective membrane area (0.0014).

- **Reverse solute flux**

$$J_{s}=\frac{{(C}_{tn}V_{tn})- {(C}_{t0}V_{t0})}{A(t_{n}-t_{0})} (S2)$$

Where J_s_ is reverse solute flux, $C_{tn}$ and $C_{t0}$ (g L^-1^) are the solute concentration at the time $t_{n}$ and $t_{0}$ (h), respectively, while V_tn_ and V_t0_ (L) are feed volume at the time $t_{n}$ and $t_{0}$, respectively, during the FO test.

- **GPC analysis**


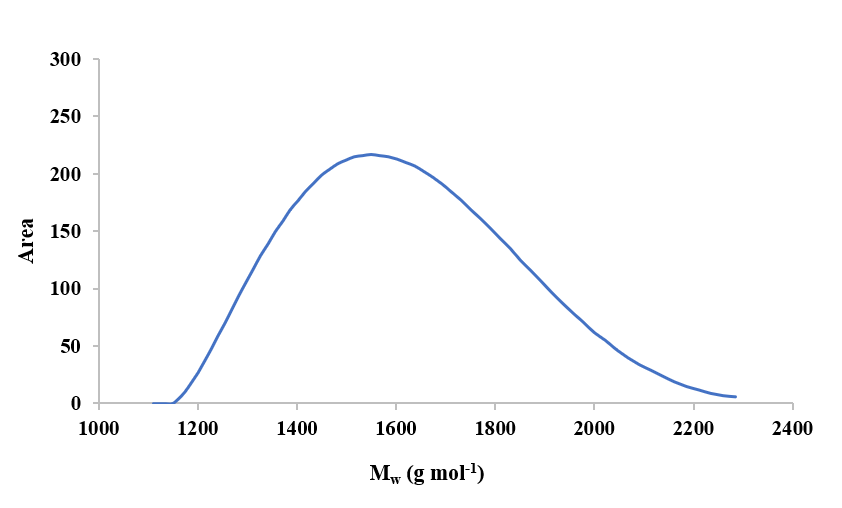


Figure S1. GPC analysis of Tween 80_1_-CC_2_ self-assembly.
